# Effect of black tea on enteral feeding tolerance in ICU patients

**Published:** 2010

**Authors:** Soheila Mojdeh, Samire Shahin, Gholamreza Khalili

**Affiliations:** * Department of Operating Room Nursing, School of Nursing and Midwifery, Isfahan University of Medical Sciences, Isfahan, Iran; ** Head Nurse of Intensive Care Unit, Alzahra Hospital, Isfahan, Iran; *** Department of Anesthesiology, School of Medicine, Isfahan University of Medical Sciences, Isfahan, Iran

**Keywords:** Camellia sinensis, enteral feeding, tea, intensive care

## Abstract

**BACKGROUND:**

Tea consumption has been known mostly as a well-drink after water in the world. Tea drink can affect balance of fluids and renal function. In addition, it can cause loss of many viruses in the stomach and can increase or decrease gastrointestinal movements. This research was done to determine the effect of tea on increasing enteral feeding tolerance in ICU patients in Alzahra Hospital.

**METHODS:**

In this clinical trial study, 45 patients were enrolled in two groups, tea consumption group and the standard method of nutrition as control group. Tea gavage was performed two times in the morning; 100 cc tea used for the study group and the same volume of water was used for the control group. Residual volume was measured before gavage. Data collected for one week. Information sheet had two pages; the first page described how to complete the form and the method of tea gavage and the second page was for data collection. Data were analyzed by t-student test, chi-square, and analysis of variance.

**RESULTS:**

In two groups, 92% of patients tolerated liquid gavage. Their difference by chi-square test was not significant. Average urine volume after black tea gavage was 783.3 L in the study group and 802.2 L in the control group. ANOVA test showed no significant difference.

**CONCLUSIONS:**

Although the difference was not statistically significant between the two groups, but in study group consumption of tea was acceptable by patients.

Tea is the most consumed drink in the after water. Iranians and a lot of enjoy a cup of tea.^1^–^3^ Black tea is good herbal plant too. Black tea, green tea and oolong tea are made from the dried leaves of *Camellia sinensis* and their differences are in production process and preparation.^4^–^6^ Black tea is a source of caffeine, a methylxanthine that stimulates the central nervous system, relaxes smooth muscles in the pulmonary airways (bronchioles), stimulates the heart, and acts on the kidney as a diuretic (increasing urine). One cup of tea contains up to 50 milligrams caffeine, depending on the strength and size of cup. Tea also contains polyphenols (catechins, anthocyanins, and phenolic acids), tannin, trace elements, and vitamins, and may reduce risk factors of heart diseases, high blood pressure, cholesterol, and blood lipids.^2^,^3^,^7^,^8^ Tea extract, depending on physiological system involvement, can increase or decrease the gastrointestinal movements; off course increase in movements are more seen.^9^ In a research about the effects of black tea extract on rat small intestine passing time, it was shown that black tea extract caused a significant increase in small bowel movements compared with the control group. The results showed that concentrations of 3 g and 4.5 g/100 ml of black tea extract significantly increased small intestine movements compared to controls while the concentration of 9 g/100 ml was not created small bowel movements.^10^ In other studies it is seen that black tea increased digestive system movements, and eliminated or less precipitated irritable bowel syndrome.^11^

The best method of feeding is through mouth, and this way is considered as the most economical and safest method of nutrition. But, in critical care units, the patients cannot eat, and when gastrointestinal system has good function, nutrition should be highly supportive;^12^ because low supportive feeding may cause increased protein break, and can delay wound healing.^13^ Enteral nutrition is generally preferred to parenteral nutrition because of its relative simplicity, safety, and low cost, as well as its ability to maintain bowel integrity in critically ill patients.^14^ Drinking a cup of tea cause loss of many viruses of the stomach^3^ and is effective on passing time of intestinal tract.^11^ On the other hand, it is harmless and has no toxicity in the short and longterm use.^6^,^5^ Thus, this survey aimed to determine the effect of black tea on enteral feeding tolerance in ICU patients.

## Methods

This study was a single blind clinical trial. Target population was ICU patients with the following inclusion criteria: be possible for enteral feeding, no history of surgery on the digestive system, no gastrointestinal (GI) bleeding, and no acute and chronic renal failure. Exclusion criteria included discharge from ICU before one week, and critical conditions such as GI bleeding and renal failure. Sampling was done randomly (odd or even number beds).

Forty five patients were participated in the study group and 45 people in the control group. Those Golestan teas (Golestan Co, Tehran, Iran) that had internal standard seals and accompanied instructions or guide lines were used. The tannin concentration determined in School of Pharmacy of Isfahan University of Medical Sciences. In this research, we used manner polls paint method and worked with Dennis balloon; 100 mg of tea powder was added to 5 ml of sterile water and shook in 5-10 minutes. The resulting solution was passed on filter paper and washed with 5 ml sterile water, and eventually 7 ml extract was obtained. To prepare the sample solutions, 1 ml of extract was added to 75 ml sterile water in three balloons. Then 5 ml Folin Denis reagent and 10 ml sodium carbonate solution were added to each balloon to create maximum possible color. Three substances were quite mixed and were quiet for 30 minutes. The sample absorption was determined by spectrophotometer on wavelength of 760 nm. Using the formula of the standard curve, the concentrations of sample tannic acid were obtained and after being multiplied by time, the amount of tannin came to weight per weight, for each sample (g/100 g tea powder). The average amount of tannin obtained from three samples. In this study, we tried that the amount of tannin that reaches the patients be less than 12.5%. In the study group we used 100 cc warm tea, and in the control group we used 100 cc water, two times in the morning for both groups. Before each gavage, stomach residual was recorded. Information sheet had two-pages; the first page described how to complete the form and the method of tea gavage and the second one was for data collection and demographic profile. Duration of data collection was one week.

The questionnaire validity was checked through content validity and the reliability was checked using test and retest. Data were analyzed by t-student, chi-square, and ANOVA tests via SPSS software.

The ethical committee of Isfahan University of Medical Sciences approved the study.

## Results

The mean (SD) age in the study and the control groups was 42(20.7) and 39(19.8) years, respectively, and t-test did not show any significant difference between them; 65(72.2%) patients were men and 25(27.8%) were wemon; 15 patients were hospitalized in central ICU and 75 patients were hospitalized in trauma ICU. The average hospitalization period was 16.4(11.6) and t-test demonstrated no significant difference between the two groups.

Average volume of liquid gavage in all patients was 183.3(111.8) ml. This volume for the study and control groups was 200.3(106.2) and 166.2(106.2) ml, respectively and t-test showed no significant difference.

Also gender factor was not effective in the lavage (aspiration of gastric fluids to determine and compare the stomach residuals) volume. In the two groups 92.2% of patients tolerated liquid gavage and 7.8% had intolerance to liquids. Mean volume of lavage liquid was measured in 6 parts of study. Average volume of lavage liquid at the beginning of study was 10.1 ml in the study group and 9.1 ml in the control group. The other mean volumes of lavage fluid were low in the study group and were up and down in the control group (Table 1).

**Table 1:** Mean and standard deviation of lavage volume in the two groups at six stages of the study

	Study group (ml)			Control group (ml)		
Time						
	mean	SD	Changes to the base time	mean	SD	Changes to the base time
Base time 8AM	10.1	2.6	0	9.1	2.6	0
10 AM	13	4.1	-2.9	6.7	2.6	2.4
12 MD	20.4	4.6	-10.3	4.9	1.9	4.2
2 PM	14.9	4.2	-4.8	8	2.3	1.1
4 PM	10.2	3.6	-0.1	4	1.7	5.1
6 PM	10	1.1	0.1	3.8	0.7	5.3

**Figure 1:**
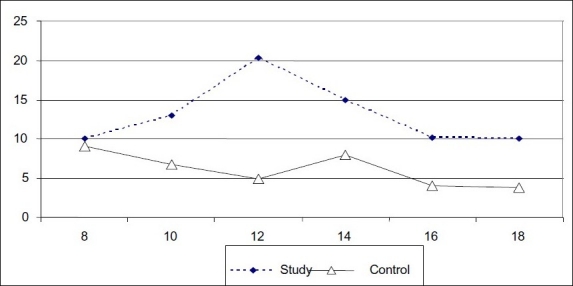
The mean volume of lavage in the two groups

The mean changes of lavage volumes were evaluated in the two groups. The highest changes of liquid lavage volume occurred in both groups at 12 MD but in opposite direction (Figure 1)

The average urine volume was measured at 12 MD, 6 PM, and total output (24 h) and ANOVA test showed no significant difference (Table 2).

**Table 2:** Mean and standard deviation of urine volumes in 6, 12 and 24 hours after gavage

Group	study (ml)			Control (ml)		
Time	mean	SD	Changes to the base time	mean	SD	Changes to the base time
6 hours after gavage	783.3	538.6	0	802.2	556.6	0
12 hours after gavage	872.2	427.5	-88.9	977.8	839.3	-175.6
24 hours after gavage	2822	1406	-2039	2963	1782	2160.8

On the other hand, ANOVA test showed no significant relationship between tolerance to fluids and volume urine. Other variables studied (gender and BMI) also were not significantly effective on urine volume

## Discussion

In this study, average stomach residual volume was low in the study group but in control group it was fluctuated and no significant difference between the two groups was seen. The highest amount of lavage was observed at 12 MD in the study group, whereas lavage was declined at same time in the control group. Jafari et al showed that 4.5 g/100 ml and 3 g/100 ml concentrations of black tea extract significantly increased small bowel movements compared to the control group but the concentration of 9g/l00 ml were not significantly changed small bowel movements. In an animal study, it was revealed that tea extracts can increase or decrease gastrointestinal movements.^9^ Another research showed that the average concentration of black tea helps relieve irritable bowel syndrome (IBS) symptoms or less stimulate digestive system in these patients.^13^ We anticipated increased bowel movements due to stomach earlier discharge, which did not occur.

Aspiration of gastric secretions showed that 92.2% patients tolerated the liquid gavage in the two groups. The results of an in vitro research showed that the recovery group was fasting for 3 days which led to villi atrophy. Ingestion of green tea and, to a lesser extent, vitamin E for 7 days helped in the recovery of villi to normal. In the pretreatment set, drinking green tea, black tea, or vitamin E for 14 days before fasting protected intestinal mucosa from damage.^15^ In addition, ingestion of black tea protected the intestinal mucosa against atrophy, but we did not do these measurements such as laboratory test or biopsy tissue. Also, Tani et al studied the effects of standardized extraction plant species on the activities of critical organs (cardiovascular, respiratory, digestive and nervous systems) in mice. The results showed that mice treated with the extract, 1 g/kg, had decreased blood pressure and stomach acid.^16^ An herbal plant, such as tea, contains tannin and has anti-hemorrhagic and anti-diarrheic activities and protects the digestive organs against damage,^17^ but we did not have circumstances that can evaluate these properties. The average urine volume was measured in three parts. The similarity of two groups was due to diuretic properties of tea. It seems that tea consumption cause the increase of urine output. This issue could reduce edematous situations in patients with complete bed rest but in ICU patients fluids intake is based on fluids output. Another research study on the effects of chronic consumption of Kombucha tea, a black tea, on weight loss in diabetic rats showed that all groups had significant weight loss. In the control group, weight loss was progressive and continued, but after 15 day consumption of Kombucha tea, progressive increase in weight, in both observational and study groups was observed. Finally, test between control and study groups showed no significant difference between them in weight change.^13^ In Iranian culture, tea has a special place. In the study group, patients were satisfied of tea consumption to the extent that after the end of the study period (one week) they requested to continue the program of tea drinking. ICU patients’ conditions are very variable and each person had his own particular circumstances. The understanding of the biological activities and health benefits of tea polyphenols is still very limited. Further indepth studies are needed to investigate the safety and efficacy of tea in humans and to determine the different mechanisms of tea in health protection.^18^

The authors declare no conflict of interest in this study.
